# Development of an Aptamer Based Luminescent Optical Fiber Sensor for the Continuous Monitoring of Hg^2+^ in Aqueous Media

**DOI:** 10.3390/s20082372

**Published:** 2020-04-22

**Authors:** Nerea De Acha, César Elosúa, Francisco J. Arregui

**Affiliations:** 1 Department of Electrical, Electronic and Communications Engineering, Public University of Navarra, Ed. Los Tejos, Campus Arrosadía s/n, E-31006 Pamplona, Navarra, Spain; 2 Institute of Smart Cities, Public University of Navarra, Ed. Jerónimo de Ayanz, Campus Arrosadía s/n, E-31006 Pamplona, Navarra, Spain

**Keywords:** optical fiber biosensor, luminescent biosensor, fluorophore-labelled aptamer, mercury detection

## Abstract

A fluorescent optical fiber sensor for the detection of mercury (Hg^2+^) ions in aqueous solutions is presented in this work. The sensor was based on a fluorophore-labeled thymine (T)-rich oligodeoxyribonucleotide (ON) sequence that was directly immobilized onto the tip of a tapered optical fiber. In the presence of mercury ions, the formation of T–Hg^2+^-T mismatches quenches the fluorescence emission by the labeled fluorophore, which enables the measurement of Hg^2+^ ions in aqueous solutions. Thus, in contrast to commonly designed sensors, neither a fluorescence quencher nor a complementary ON sequence is required. The sensor presented a response time of 24.8 seconds toward 5 × 10^−12^ M Hg^2+^. It also showed both good reversibility (higher than the 95.8%) and selectivity: the I_0_/I variation was 10 times higher for Hg^2+^ ions than for Mn^2+^ ions. Other contaminants examined (Co^2+^, Ag^+^, Cd^2+^, Ni^2+^, Ca^2+^, Pb^2+^, Mn^2+^, Zn^2+^, Fe^3+^, and Cu^2+^) presented an even lower interference. The limit of detection of the sensor was 4.73 × 10^−13^ M Hg^2+^ in buffer solution and 9.03 × 10^−13^ M Hg^2+^ in ultrapure water, and was also able to detect 5 × 10^−12^ M Hg^2+^ in tap water.

## 1. Introduction

Assuring a good quality of water is essential in order to guarantee the health of the population and the quality of the environment [1]. Thus, public institutions are increasingly concerned about monitoring the presence of different water pollutants such as plastics [2], pesticides [3], or metal ions [4] in aqueous media, even at trace levels. Among the different metal ions, mercury (Hg^2+^) is known to be one of the most toxic species of aqueous media [5]. Apart from being related to carcinogenic processes and neurodegenerative illnesses [6], Hg^2+^ ions present an accumulative effect in the human body [7] and are non-degradable [8], so their monitoring is crucial in order to assure a good quality of water. In fact, the European Union (EU) has established the maximum allowable mercury concentration in surface water as 3.49 × 10^−10^ M [9]. As a consequence, the detection of this water contaminant has attracted the interest of scientists during the last few years, leading to the development of a wide variety of Hg^2+^-sensitive devices [10,11,12].

It is well known that a key parameter of sensors is their selectivity [13], thus, currently, aptamers have been widely utilized as these single-stranded oligonucleotides have the capability of binding to certain molecules with high affinity and specificity, so they provide a high selectivity [14]. In particular, due to the high reactivity that thymine (T) presents for Hg^2+^ ions [15], T-rich oligodeoxyribonucleotide (ON) sequences have been used for Hg^2+^ sensing purposes [16], presenting low cross-sensitivity values for other metal ions. For instance, Li et al. [17] reported a device that showed negligible interference from K^+^, Ag^+^, Ca^2+^, Cd^2+^, Co^2+^, Cu^2+^, Fe^3+^, Ni^2+^, and Pb^2+^: even when their concentrations were four-fold higher than that of Hg^2+^ (200 nM of other metal ions against 50 nM of Hg^2+^), the sensor response barely changed. Further examples of aptamer-based sensors that exhibit high selectivity were analyzed in detail in [18]. Furthermore, T–Hg^2+^–T has the capability of absorbing the electrons emitted by a fluorophore labelled to one of the termini of the ON sequences [19], causing a quenching of the emitted intensity [20]. This fact entails several advantages over the Hg^2+^ sensors developed to date: first, there is no need to utilize any complementary ON sequence (thus, the Hg^2+^ ion concentration of the sample is not altered), which usually happens with this kind of sensor [21]; and second, no quencher has to be labelled to the opposite termini of the same ON [22], which enables the sensitive ON sequence to be directly immobilized onto a substrate.

Among the different substrates that can be employed for the fabrication of these sensors, optical fiber can be considered as one of the most suitable. As it is made of silica, the chemical robustness and lack of oxidation of this material enables it to be placed in aqueous media for long periods of time [23]. Furthermore, optical fiber sensors do not require referenced measurements like pH-meters or ion-meters [24].

Another advantage that optical fiber offers is that silica surfaces can be easily modified in order to allow the deposition of different materials such as polymers [25], polyelectrolytes [26], or even biochemical species [27]. Furthermore, the properties of the deposited materials can be tailored in a nanometric scale [28], which enables the fabrication of custom-made sensors. Moreover, optical fiber shows interesting features for the development of luminescence-based sensors: it permits a robust and simple experimental set-up where the light source, the sensor, and spectrometer are directly connected, in which the excitation and the luminescence signals are transmitted through the same fiber [29]. Additionally, the optical fiber tip can be shaped to couple as much light as possible [30].

As has been previously explained, monitoring Hg^2+^ ions at trace levels is relevant. In addition, T-rich ON sequences show interesting features for the detection of these ions: they present high affinity toward them, and, furthermore, the T–Hg^2+^–T mismatches can quench the fluorescence emissions. Thus, in this work, a fluorophore labeled ON sequence was immobilized onto the tapered end of an optical fiber. The behavior of the sensor in terms of sensitivity, reversibility, and cross-sensitivity was analyzed in a phosphate buffered solution (PBS). To check out the further applicability of the sensor, Hg^2+^ ions detection was also studied in ultrapure water and tap water. 

## 2. Materials and Methods

### 2.1. Chemicals and Reagents for the Fabrication of the Sensor

Due to the high affinity of thymine (T) rich ON sequences for Hg^2+^ ions [15], in this work the HPLC-grade oligodeoxyribonucleotide sequence 5′-NH_2_-(CH_2_)_6_–TTCTTTCTTCGCGTTGTTTGTT–Atto390–3′ was used as the Hg^2+^-sensitive material (purchased from Metabion (Planegg, Germany)). For its storage, a 50 μM probe stock solution was prepared in a 10 mM phosphate buffered solution (PBS) (acquired from Merck (Darmstadt, Germany)) at pH 7.4, which was kept frozen at −22 °C in the absence of light.

The utilization of this sequence involves a key advantage over other ON sequence-based Hg^2+^ sensors, as the T–Hg^2+^–T mismatches absorb the electrons emitted by the labelled atto390 (consequently quenching the fluorescent emission), there is no need to use either a quencher [31] or a complementary sequence [32]. This also allows the sequence to become directly attached onto the optical fiber, as it is explained hereafter.

The cleaved end of a fiber pigtail was modified to increase the interface area and therefore the coupled luminescent emission. To achieve this, a taper was shaped on the tip of the optical fiber with hydrofluoric acid (HF) (48% purity); the surrounding cladding was removed with ethanol (99.8% purity). Both were bought from Panreac (Barcelona, Spain).

Thereafter, the cleaning and activation processes of the optical fiber surface were performed using a piranha solution (H_2_SO_4_/H_2_O_2_, 3:1 (v/v), acquired at Sigma Aldrich (Darmstadt, Germany) and Panreac (Barcelona, Spain), respectively).

For the immobilization of the Hg^2+^-sensitive ON sequence on the surface of the optical fiber, (3-aminopropyl)trimethoxysilane (97%) (APTMS), methanol (99.8% purity), and glutaraldehyde (GA) (25% aqueous solution) were utilized. These were purchased from Sigma Aldrich (Darmstadt, Germany) and used as received. 

### 2.2. Taper Fabrication

In order to couple as much luminescence emission as possible from the fluorophore-labeled ON sequence to the optical fiber, it was immobilized onto the tapered end of a 1000 μm-core plastic cladding silica fiber [33,34]. The length of the taper was 3 mm, and it was fabricated by the subsequent immersion and withdrawal of the tip of the optical fiber in and out of a 48% (v/v) hydrofluoric acid solution at constant speeds of 100 mm/min and 2 mm/min, respectively. This procedure was repeated 40 times and afterward, the cladding of the fiber was removed with a flame and the resulting taper was cleaned with ethanol.

### 2.3. Immobilization of the ON Sequence onto the Optical Fiber

**Figure 1:**
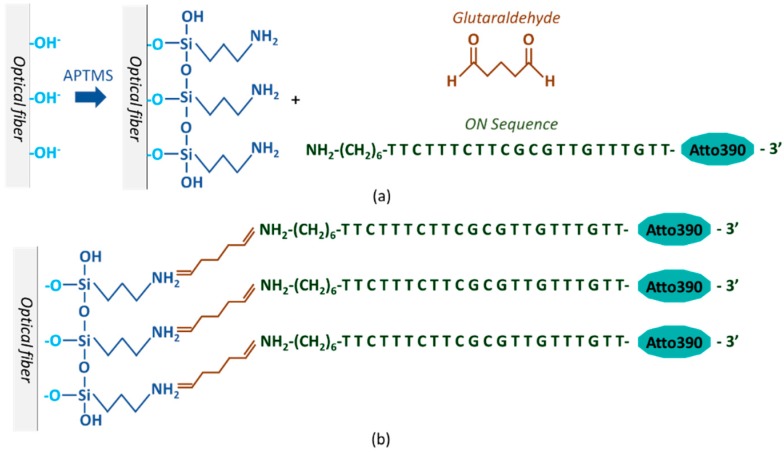
(**a**) Immobilization process of the ON sequence onto the surface of the optical fiber: first, the OH^−^ groups were activated with piranha solution, then the surface was silanized with APTMS and finally, the ON sequence was cross-linked utilizing glutaraldehyde. (**b**) Schematic of the sensing structure.

The immobilization procedure of the ON sequence onto the tapered end of the fiber, which is shown in Figure 1, consisted of the following steps [35]: first, the cleaning and OH^−^ group activation processes of the fiber surface were carried out by immersing for 10 min in the piranha solution, followed by a thorough wash with ultrapure water. For the amination of the optical fiber, it was kept for 16 h in a 2% (v/v) APTMS solution in methanol, then washed with methanol, and dried for an hour at 110 °C afterwards. Subsequently, the aminated fiber was dipped for an hour in a GA solution (10% (v/v) in PBS (pH 8.5)) at 21 °C and washed with ultrapure water. Finally, the ON sequence was adsorbed onto the fiber by immersing it for 2 hours in a 250 nM aptamer solution in 10 mM PBS (pH 7.4) mixture and washed with 10 mM PBS (pH 7.4). All sensors were stored at 4 °C in the absence of light and were fabricated following this procedure.

### 2.4. Preparation of the Samples for Hg^2+^ Analysis

Since the first part of this study consisted of the detection of Hg^2+^ ions in 10 mM PBS (pH 7.4) solution, a 10^−2^ M Hg^2+^ stock solution in PBS was prepared. This solution was subsequently diluted to obtain 5 × 10^−12^ M Hg^2+^, 10^−11^ M Hg^2+^, 5 × 10^−11^ M Hg^2+^, 10^−10^ M Hg^2+^, 5 × 10^−10^ M Hg^2+^, 10^−9^ M Hg^2+^, and 5 × 10^−9^ M Hg^2+^ solutions in PBS (pH 7.4). The latter was the highest Hg^2+^ ion concentration analyzed because higher ones were not considered of interest, as the maximum Hg^2+^ concentration allowed by the EU in surface water is 3.49 × 10^−10^ M [9].

A similar procedure was carried out in order to achieve the different concentrations of Hg^2+^ ions in ultrapure water and in tap water. In order to regenerate the sensing film, a 0.5% (w/w) sodium dodecyl sulfate (SDS) [36] (Sigma Aldrich (Darmstadt, Germany)) solution was prepared.

Finally, with the aim of analyzing the cross-sensitivity of the sensor toward other metal ions, 10^−6^ M solutions of Co^2+^, Ag^+^, Cd^2+^, Ni^2+^, Ca^2+^, Pb^2+^, Mn^2+^, Zn^2+^, Fe^3+^, and Cu^2+^ in PBS (pH 7.4) were respectively obtained from standard solutions of Co(NO₃)₂, AgNO₃, Cd(NO_3_)_2_, Ni(NO_3_)_2_, Ca(NO_3_)_2_, Pb(NO_3_)_2_, Mn(NO_3_)_2_, Zn(NO_3_)_2_, Fe(NO_3_)_3_ and Cu(NO₃)₂ in 0.5 mol/l of nitric acid. All were purchased from Merck (Darmstadt, Germany).

### 2.5. Sensing Mechanism

**Figure 2:**
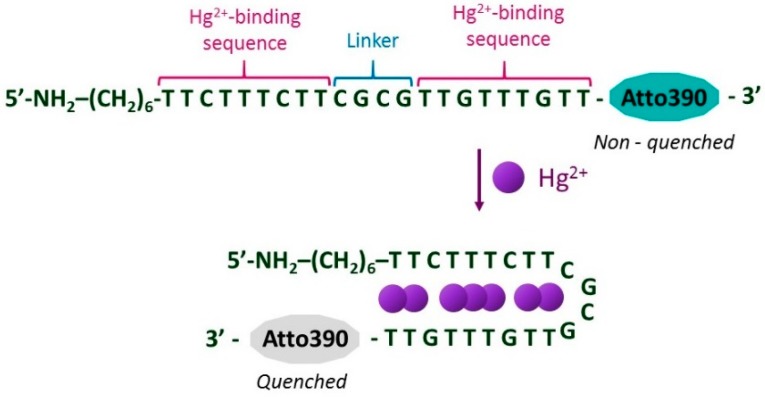
Schematic representation of the hairpin structure acquired by the ON sequence in the presence of Hg^2+^ ions due to the formation of T–Hg^2+^–T mismatches, which also quenches the luminescence emission of the fluorophore Atto390.

T-rich ON sequences are well-known for their high affinity toward Hg^2+^ ions [15]: in particular, in the presence of this metal ion, T–Hg^2+^–T mismatches are formed [37], causing the ON sequence to acquire a hairpin structure [38], as depicted in Figure 2. Furthermore, in the case of fluorophore-labeled ON sequences, there was an electron transfer from the excited fluorophore to the T–Hg^2+^–T pairs [19], which was the cause of the decrease in the luminescent emission of the fluorophore [20]. This fact has been widely employed to develop luminescent Hg^2+^ ion sensors that use ON sequences [39].

As SDS is a chemical denaturant, it has been commonly used for the regeneration of this kind of sensor [35]: the hydrocarbon tail of the surfactant binds to the ON sequence [40]. This provokes the dissociation of the T–Hg^2+^–T mismatches and restores the sensor response; when these structures are disjointed, there is no absorption of the luminescent emission, so it recovers its original intensity.

### 2.6. Characterization of the Sensors

In order to analyze the response of the sensor to distinct Hg^2+^ ion concentrations in aqueous solutions, it was first exposed to PBS (pH 7.4) mixtures with different Hg^2+^ concentrations. The characterization was performed as follows:

- First, the sensor was exposed to the continuous illumination of the LED for at least 90 minutes to estimate the mathematical characterization of the photobleaching suffered by the fluorophore labelled to the ON sequence [41]. During this test, the sensor was in a PBS (pH 7.4) solution.
- Hereafter, it was exposed for five minutes to a PBS (pH 7.4) solution with a certain Hg^2+^ concentration and then regenerated by dipping it in a 0.5% (w/w) SDS solution.
- Afterward, the sensor was introduced again into a PBS (pH 7.4) solution for 20 minutes until the fluorescence became stabilized.

The behavior of the sensor toward Hg^2+^ (in terms of luminescent intensity and kinetics) was analyzed by repeating this procedure successively with different concentrations of Hg^2+^ ions in PBS (pH 7.4) solutions. The detection of Hg^2+^ in ultrapure (or tap) water was carried out in a similar procedure, only replacing the PBS solution with ultrapure or tap water.

Regarding the luminescence measurements, in order to avoid any kind of fluctuation of the luminescent intensity due to undesired artifacts of the light source [41], all of the recorded spectra were normalized to the LED averaged intensity at 390 nm.

The calibration curves of the sensors in each media were obtained by dividing the normalized luminescence intensity of each sensor in the absence of Hg^2+^ ions (I_0_) by the normalized intensity of the sensor for the different Hg^2+^ concentrations (I) [42].

## 3. Experimental Set-Up

**Figure 3:**
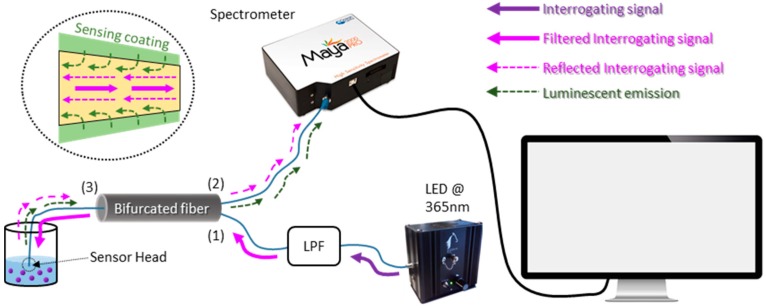
Experimental set-up utilized to characterize the sensor: the blue lines correspond to the optical fibers, while the black one represents the USB cable. (Inset) A scheme of the sensor tip.

Due to the nature of the light signal and to have a robust experimental set-up, an optimized reflection architecture was employed (see Figure 3) [41]. The configuration was based on the utilization of a 600 μm-core bifurcated fiber (acquired at Ocean Insight (Ostfildern, Germany)): the sensor was connected to the common branch of this fiber (3), whereas the LSL Maya spectrometer (from Ocean Insight (Ostfildern, Germany)) and a LED centered at 365 nm (purchased from Pyroistech (Pamplona, Spain)) were connected to branches (1) and (2) of the bifurcated fiber, respectively. The interrogating signal from the source was coupled via branch (1) toward the sensor head where it excited the luminescent emission of the sensing material (modulated by the Hg^2+^ ion concentration); on the other hand, the sensor response was guided backward through branch (2) to the spectrometer. The luminescent emission of the Atto390 (centered at 460 nm) required a high optical power that was also reflected in the sensor tip and could overlap the sensing signal. To reduce this effect, on one hand, the LED was connected through a 200 μm-core optical fiber to an adjustable low pass filter (LPF) from Ocean Optics, with its cut-off wavelength at 390 nm, and then to branch (1); on the other hand, the stretched end of the sensor (described in Section 2.2) reduced the reflected interrogating signal. All the spectra were registered by the software OceanView 1.0 (Ocean Insight (Ostfildern, Germany)) using an integration time of 1500 ms.

## 4. Results and Discussion

### 4.1. Detection of Hg^2+^ Ions in Phosphate Buffered Solution (PBS, pH 7.4) Solutions

The behavior of the sensor toward Hg^2+^ was first analyzed in 10 mM PBS (pH 7.4) solutions. The Hg^2+^ concentration studied ranged three and a half orders of magnitude. To have a representative characterization along this span, the concentrations chosen were the limits of each decade and the middle point between them: 5 × 10^−12^ M Hg^2+^, 10^−11^ M Hg^2+^, 5 × 10^−11^ M Hg^2+^, 10^−10^ M Hg^2+^, 5 × 10^−10^ M Hg^2+^, 10^−9^ M Hg^2+^, and 5 × 10^−9^ M Hg^2+^. The sensor was regenerated with 0.5% (w/w) SDS afterward and then dipped for 20 minutes into PBS solution with no metallic ions. 

**Figure 4:**
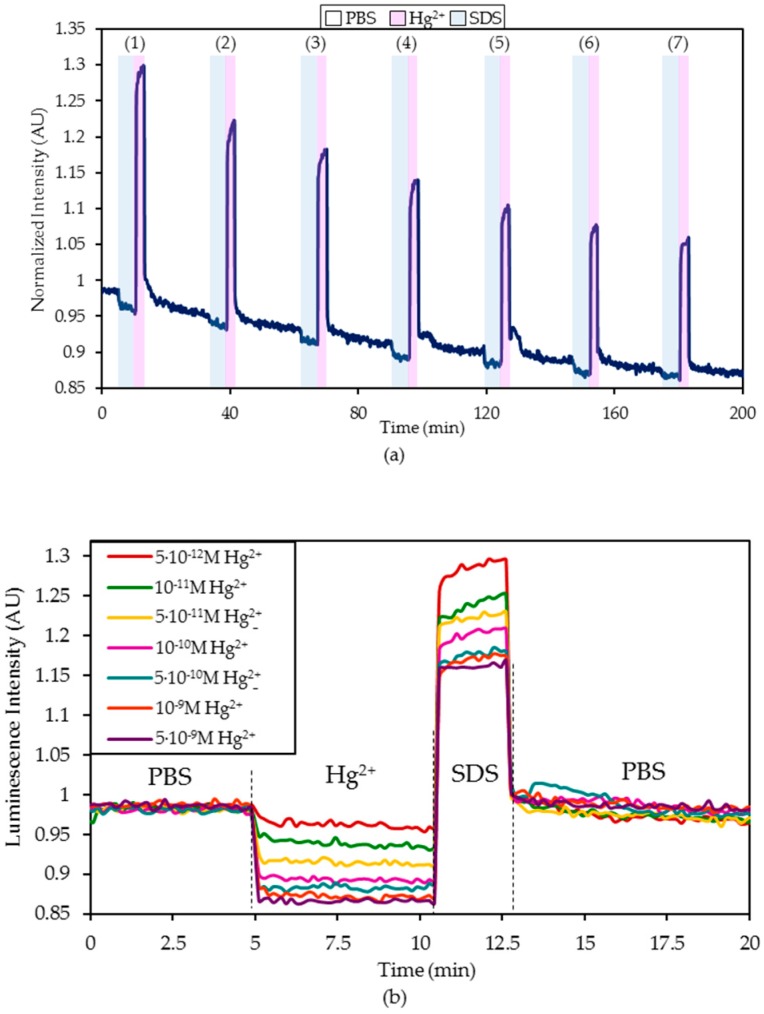
(**a**) Dynamic variation of the normalized luminescence intensity under 5 min exposure to different Hg^2+^ concentrations ((1) 5 × 10^−12^ M, (2) 10^−11^ M, (3) 5 × 10^−11^ M, (4) 10^−10^ M, (5) 5 × 10^−10^ M, (6) 10^−9^ M, (7) 5 × 10^−9^ M), 2 min regeneration with 0.5% w/w SDS and 20 min stabilization in phosphate buffered solution (PBS) (pH 7.4). (**b**) Detail of the intensity variation for each Hg^2+^ concentration in PBS buffer.

The dynamic variation of the normalized luminescence intensity for these concentrations is displayed in Figure 4: as can be observed, the emitted intensity immediately decreased in the presence of a 5 × 10^−12^ M (and higher) Hg^2+^ ion concentration and increased while the sensor was being regenerated with 0.5% (w/w) SDS. Once the sensor was immersed again in PBS, the luminescence recovered between 95.8% and 99.17% of its original value. This kind of drift in the baseline is commonly reported in the bibliography and can be mathematically compensated [43,44]. In this specific case, these intensity variations during and after the immersion of the sensor in SDS might be explained by the effect of this compound on the ON sequence: first, SDS induced an unfolding of the sequence [45] and this conformational change led to an increase in the fluorescence intensity (Figure 4a). Second, due to the unfolding of the ON sequence, the bonds between Hg^2+^ and T groups were broken, thus, the recovery values between 95.8% and 99.17% implied that not all of the T–Hg^2+^–T mismatches were fragmented. 

Looking to ensure that the Hg^2+^ ions were interacting with the ON sequence and there were no other interfering parameters, the refractive indices [35] and pH values [46] of the different Hg^2+^ solutions were checked. All of them presented similar refractive indices and pH values to the reference solution (PBS, pH 7.4), as can be observed in Supplementary Table S1.

Figure 4b shows the zoomed response before, during, and after the exposure to the different Hg^+2^ concentrations to ease the comparison between them. All the signal variations were referenced to the intensity level of the first immersion into PBS. The response time for the sensor was faster as the Hg^2+^ concentration increased: for the slowest case measured with 5 × 10^−12^ M Hg^2+^, it was 24.8 seconds.

**Figure 5:**
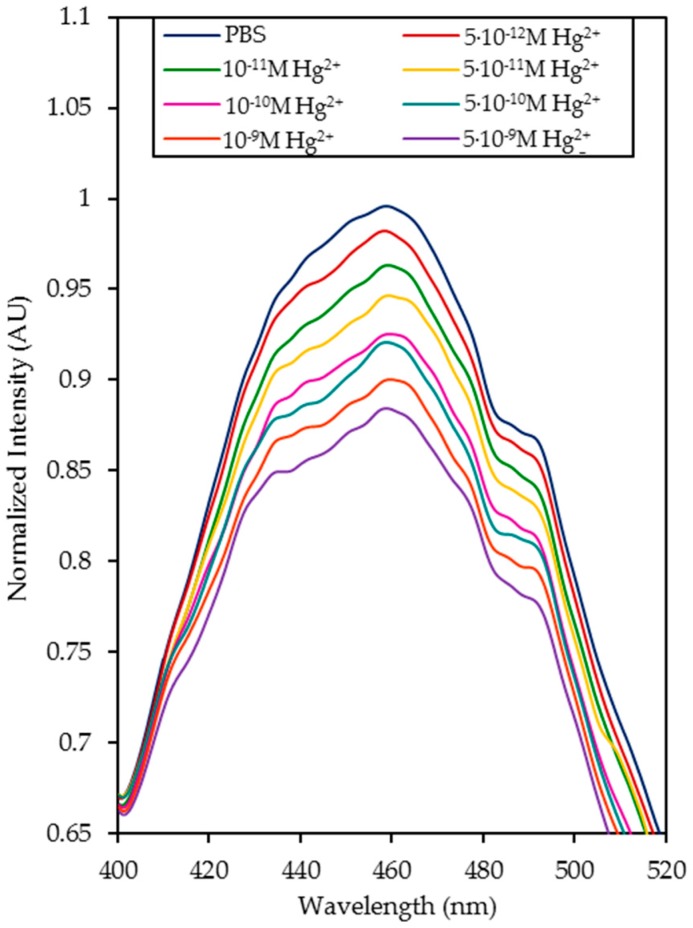
Normalized luminescence intensity for different Hg^2+^ concentrations: 0 M, 5 × 10^−12^ M, 10^−11^ M, 5 × 10^−11^ M, 10^−10^ M, 5 × 10^−10^ M, 10^−9^ M, and 5 × 10^−9^ M.

**Figure 6:**
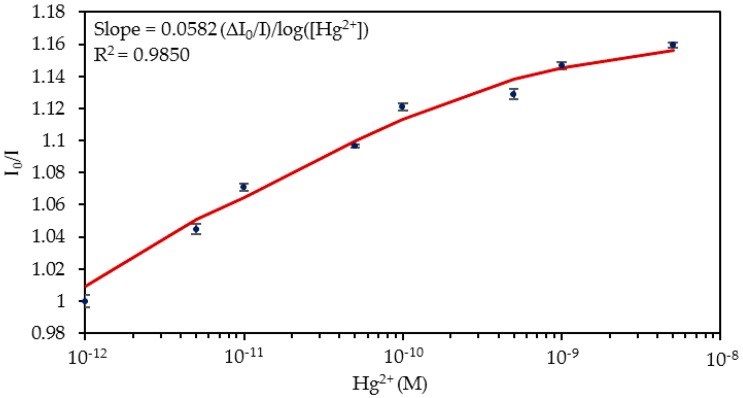
I_0_/I ratio for the different Hg^2+^ concentrations in PBS solution. The horizontal axis is shown in logarithmic scale.

The normalized luminescence peaks for each concentration are shown in Figure 5 and the calibration curve of the sensor is displayed in Figure 6. Considering these data, it can be estimated that the limit of detection (LOD) [47] was 4.73 × 10^−13^ M Hg^2+^. Its dynamic range was from 5 × 10^−12^ M to 10^−10^ M Hg^2+^, and it was saturated with concentrations higher than 5 × 10^−9^ M Hg^2+^. The sensitivity expresses the relationship between the variation in the I_0_/I ratio and the logarithm of Hg^2+^ concentration, whose value was 0.0582 Δ(I_0_/I)/log (Hg^2+^), R^2^ = 0.9850.

### 4.2. Detection of Hg^2+^ Ions in Ultrapure Water

**Figure 7:**
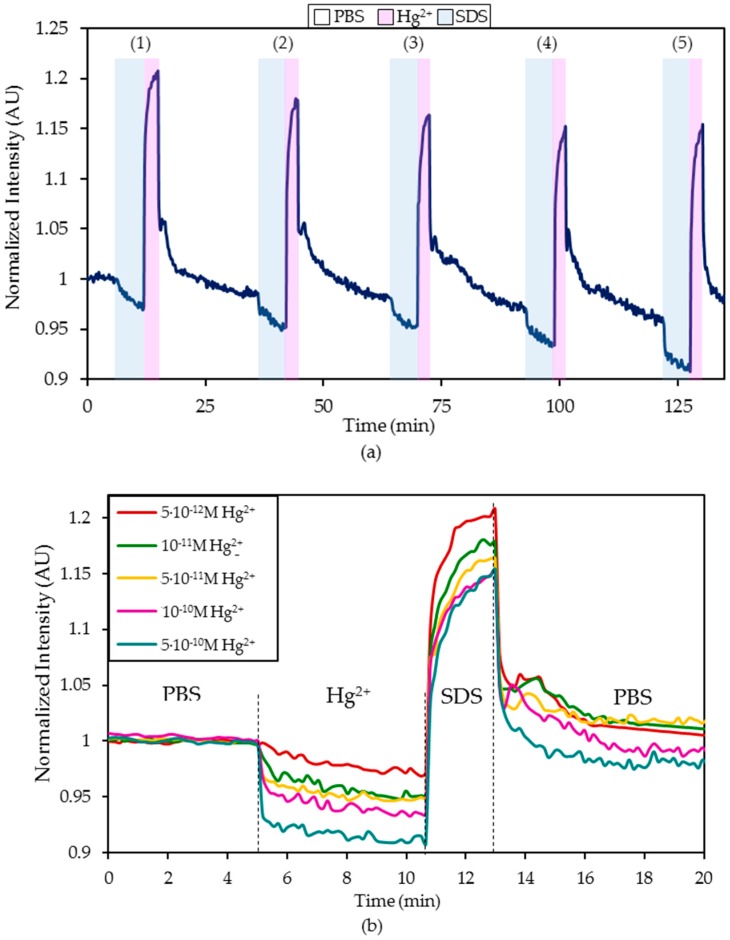
(**a**) Dynamic variation of the normalized luminescence intensity after 5 min exposure to different Hg^2+^ concentrations ((1) 5 × 10^−12^ M, (2) 10^−11^ M, (3) 5 × 10^−11^ M, (4) 10^−10^ M, (5) 5 × 10^−10^ M, (6) 10^−9^ M, (7) 5 × 10^−9^ M), 2 min regeneration with 0.5% w/w SDS and 20 min stabilization in ultrapure H_2_O. (**b**) Detail of the intensity variation for each Hg^2+^ concentration in ultrapure water.

**Figure 8:**
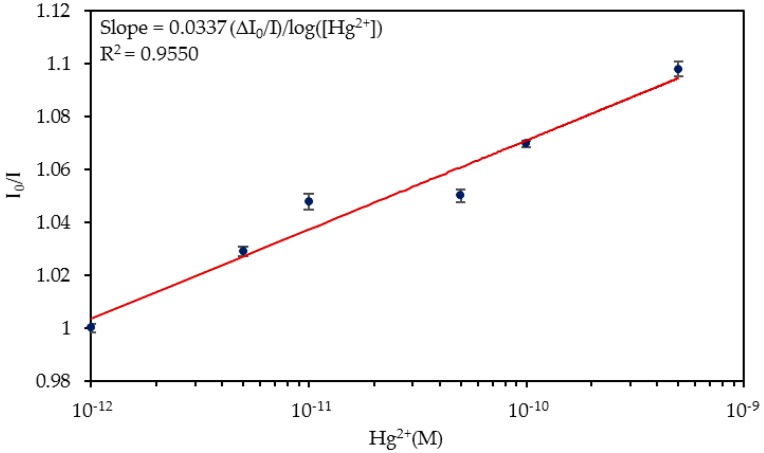
I_0_/I ratio for the different Hg^2+^ concentrations in ultrapure water. The horizontal axis is shown in logarithmic scale.

In order to analyze the feasibility of using this sensor in a real application, the detection of Hg^2+^ ions in ultrapure water was studied. As well as in the previous case, the sensor was exposed to an increasing Hg^2+^ concentration series (from 5 × 10^−12^ M Hg^2+^ to 5 × 10^−10^ M Hg^2+^) in ultrapure water, regenerating the probe with a 0.5% (w/w) SDS solution and then immersed in ultrapure water until the luminescent intensity was stabilized. The dynamic variation of the normalized luminescence is displayed in Figure 7a and detailed in Figure 7b. The resulting I_0_/I ratios are shown in Figure 8. As observed in the PBS solutions, the dynamic response of the sensor ranged from 5 × 10^−12^ M to 10^−10^ M Hg^2+^. The obtained LOD value was 9.03 × 10^−13^ M Hg^2+^, which was slightly higher than in the case of detecting Hg^2+^ ions in 10 mM PBS (pH 7.4) solution.

The lower sensitivity 0.0337 Δ(I_0_/I)/log(Hg^2+^), (R^2^ = 0.9550) that the sensor presented in ultrapure water could be explained by the pH of the solutions: while in the previous study the pH value of the reference (PBS) and the Hg^2+^ solutions was 7.4, in this case, the pH values of the different solutions varied from 5.7 and 6, whereas that of the ultrapure water was 4.94 (Supplementary Table S2). First, it must be taken into account that ON sequences are stable in the pH range 7.0–8.0 and second, that the pH value can affect the structure of the binding sequence, being maximal at pH 7.5 and lower at different pH values [46,48]. Although the pH range of the water was not the optimum for the operation of the ON sequence, the sensor presented a proper and repetitive response to the different concentrations of Hg^2+^ ions.

### 4.3. Detection of Hg^2+^ Ions in Tap Water

**Figure 9:**
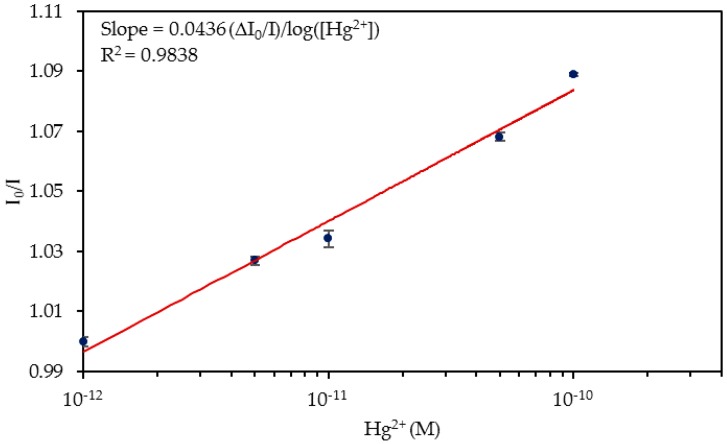
I_0_/I ratio for the different Hg^2+^ concentrations in tap water. The horizontal axis is shown in logarithmic scale.

The sensor was also exposed to tap water samples spiked with different Hg^2+^ concentrations (from 5 × 10^−12^ M Hg^2+^ to 5 × 10^−10^ M Hg^2+^). As it did not respond to dynamic changes in the Hg^2+^ concentration, it was decided to investigate the possibility of utilizing these sensors as single-use probes. Different devices were exposed to the Hg^2+^ concentrations as previously mentioned. The I_0_/I ratio of each sensor exposed to a different concentration is shown in Figure 9.

In this case, the sensitivity of 0.0436 Δ(I_0_/I)/log(Hg^2+^) (R^2^ = 0.9838) was higher than those exhibited in ultrapure water and slightly lower than the ones shown in the PBS solution. This fact could also be explained by the pH value of the solutions, which was 7.85 in the absence of Hg^2+^ ions and 7.9 in their presence (values shown in Supplementary Table S3). These pH values, although not being the optimal ones for sensing with aptamers [49], were closer than the pH values of the ultrapure water to that value. Table 1 shows the sensitivities obtained depending on the Hg^2+^ aqueous media.

A possible solution for the problems above-mentioned could be the dilution of the tap water samples into a PBS buffer. This fact, which will be analyzed in further research, would avoid the effect of the pH value on the response of the aptamers.

### 4.4. Study of the Cross-Sensitivity

**Figure 10:**
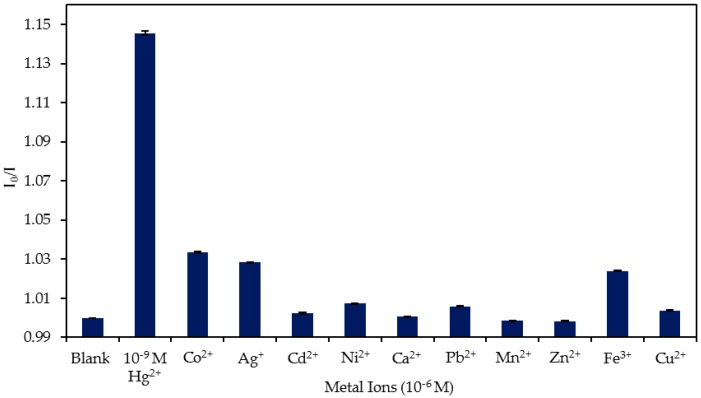
Comparison of the I_0_/I ratio of the sensor in the absence of metal ions (blank), in the presence of 10^−9^ M Hg^2+^ and in the presence of 10^−6^ M of Co^2+^, Ag^+^, Cd^2+^, Ni^2+^, Ca^2+^, Pb^2+^, Mn^2+^, Zn^2+^, Fe^3+^, and Cu^2+^.

As good selectivity is a key feature for this sensor, the device was exposed to 10^−6^ M concentrations of other metal ions (Co^2+^, Ag^+^, Cd^2+^, Zn^2+^, Ni^2+^, Ca^2+^, Pb^2+^, Mn^2+^, Fe^3+^, and Cu^2+^) as well as to 10^−9^ M Hg^2+^. The results of the test are displayed in Figure 10. Although present in a concentration 10^3^ higher than that of Hg^2+^, the other metallic ions barely interacted with the sensor. While 10^−6^ M of Cd^2+^, Ni^2+^, Ca^2+^, Pb^2+^, and Cu^2+^ induced a slight decrease in the luminescence (I_0_/I = 1.002, 1.007, 1.0004, 1.005 and 1.004, respectively), Mn^2+^ and Zn^2+^ caused a minor increment (I_0_/I = 0.998 for Mn^2+^ and I_0_/I = 0.998 for Zn^2+^) in the luminescent emission. Fe^3+^, Ag^+^, and Co^2+^ were the most interfering ions: in these cases, the sensor showed I_0_/I values of 1.04, 1.028, and 1.034, respectively. In each case, for an ion concentration 10^3^ times higher than that of Hg^2+^, the variation of the signal was five times lower than that induced by 10^−9^ M Hg^2+^. This result showed the low cross-sensitivity of the sensor toward other metallic ions.

## 5. Conclusions

A turn-off fluorescent sensor for the detection of Hg^2+^ ions was fabricated with a fluorophore-labeled T-rich ON sequence, which enabled its direct immobilization onto the tapered end of an optical fiber. Furthermore, there was no need to use a fluorescence quencher or a complementary sequence as the T–Hg^2+^–T mismatches formed in the presence of that metal ion attenuated the emitted luminescence. The sensor was capable of continuously monitoring dynamic variations in the Hg^2+^ concentration and presented a LOD of 4.73 × 10^−13^ M Hg^2+^ in PBS buffer, moreover, the base line was recovered at values that ranged from 95.8% to 99.17%. Moreover, the sensor showed an almost negligible cross-sensitivity toward other metal ions and was also able of detecting 5 × 10^−12^ M Hg^2+^ ions in ultrapure and tap water, although the pH should be adjusted to physiological values. Future research lines should also analyze the effect of the ON-sequence length on the sensitivity and limit of detection of the sensor.

## Figures and Tables

**Table 1:** Sensitivities measured for the different aqueous solutions.

Media	Sensitivity Δ(I_0_/I)/log([Hg^2+^])	R^2^
PBS solutions	0.0582	0.9850
Ultrapure water	0.0337	0.9550
Tap Water	0.0436	0.9838

## Supplementary Materials

The following are available online at https://www.mdpi.com/1424-8220/20/8/2372/s1, Table S1: Measurement of the pH values and refractive indices of the Hg^2+^ solutions in PBS; Table S2: Measurement of the pH values and refractive indices of the Hg^2+^ solutions in ultrapure water; Table S3: Measurement of the pH values and refractive indices of the Hg^2+^ solutions in tap water.
