# Supplementary material for: Development of an Aptamer Based Luminescent Optical Fiber Sensor for the Continuous Monitoring of Hg2+ in Aqueous Media
Source: Sensors (Basel). 2020 Apr 22;20(8):2372. doi: 10.3390/s20082372 (PMC7219322; doi:10.3390/s20082372)
Supplement: Supplementary file 1 [file sensors-20-02372-s001.pdf]

sensors

MDPI

Article

Development of an Aptamer Based Luminescent Optical Fiber Sensor for the Continuous Monitoring of Hg^{2+} in Aqueous Media

Nerea De Acha [1,*], César Elosúa [1,2] and Francisco J. Arregui [1,2]

1 Department of Electrical, Electronic and Communications Engineering, Public University of Navarra, Ed. Los Tejos, Campus Arrosadía s/n, E-31006 Pamplona, Navarra, Spain
2 Institute of Smart Cities, Public University of Navarra, Ed. Jerónimo de Ayanz, Campus Arrosadía s/n, E-31006 Pamplona, Navarra, Spain
* Correspondence: nerea.deacha@unavarra.es; Tel: +34-948-166-044

Table 1. Measurement of the pH values and refractive indices of the Hg^{2+} solutions in PBS.

Hg^{2+} (M)	pH	Average RI
0 (PBS)	7.44	1.33456
5×10^{-12}	7.44	1.33454
10^{-11}	7.46	1.33456
5×10^{-11}	7.44	1.33454
10^{-10}	7.49	1.33454
5×10^{-10}	7.42	1.33458
10^{-9}	7.47	1.33448
5×10^{-9}	7.41	1.33466

Table 2. Measurement of the pH values and refractive indices of the Hg^{2+} solutions in ultrapure water.

Hg^{2+} (M)	pH	Average RI
0 (ultrapure H_2O)	4.94	1.3333
5×10^{-12}	5.98	1.33326
10^{-11}	5.93	1.33312
5×10^{-11}	5.74	1.33334
10^{-10}	5.71	1.33288
5×10^{-10}	5.8	1.33322

Table 3. Measurement of the pH values and refractive indices of the Hg^{2+} solutions in tap water.

Hg^{2+} (M)	pH	Average RI
0 (tap H_2O)	7.85	1.3332
5×10^{-12}	7.89	1.33332
10^{-11}	7.89	1.33326
5×10^{-11}	7.91	1.33326
10^{-10}	7.88	1.33324
